# Comparative Study of Automated Real-Time Left Atrial Appendage Sizing Using Patient-Specific 3D Heart Models Versus Transesophageal Echocardiography and Multidetector Computed Tomography in Patients with Nonvalvular Atrial Fibrillation: Implications for Device Selection

**DOI:** 10.3390/jcm14165696

**Published:** 2025-08-12

**Authors:** Dominika Kanschik, Amin Polzin, Houtan Heidari, Lisa Dannenberg, Raphael Phinicarides, Kathrin Klein, Nikos Werner, Malte Kelm, Christian Jung, Tobias Zeus, Shazia Afzal

**Affiliations:** 1 Department of Cardiology, Pneumology, and Vascular Medicine, University Hospital Duesseldorf, 40225 Dusseldorf, Germany; dominikaanna.kanschik@med.uni-duesseldorf.de (D.K.); amin.polzin@med.uni-duesseldorf.de (A.P.); houtan.heidari@med.uni-duesseldorf.de (H.H.); lisa.dannenberg@med.uni-duesseldorf.de (L.D.); raphael.phinicarides@med.uni-duesseldorf.de (R.P.); kathrin.klein@med.uni-duesseldorf.de (K.K.); malte.kelm@med.uni-duesseldorf.de (M.K.); christian.jung@med.uni-duesseldorf.de (C.J.); 2 Cardiovascular Research Institute Duesseldorf (CARID), Medical Faculty, Heinrich-Heine University, 40225 Duesseldorf, Germany; 3 Heartcenter Trier, Krankenhaus der Barmherzigen Brueder, 54292 Trier, Germany; n.werner@bbtgruppe.de (N.W.); s.afzal@bbtgruppe.de (S.A.)

**Keywords:** left atrial appendage closure, fusion imaging, artificial intelligence, 3D echocardiography, structural heart disease

## Abstract

**Background:** An accurate device sizing for percutaneous left atrial appendage closure (LAAC) can be challenging. Intraprocedural automated LAA evaluation by transoesophageal echocardiography (TEE) based on an artificial intelligence-assisted 3D model may facilitate sizing and prediction of C-arm angulation for device implantation in a one-stop-shop procedure. This study aimed to evaluate the feasibility and accuracy of automated echocardiographic LAA sizing based on a patient-specific 3D heart model prototype in real-time. **Methods:** A prospective monocentric study was conducted in 66 patients who underwent LAAC with the Amulet device. All major LAA morphologies were represented. Preprocedural multidetector computed tomography (MSCT) measurements and intraprocedural TEE and angiography measurements of the ostium, landing zone, and C-arm angulation were compared with the 3D heart model measurements. **Results:** The procedure achieved a 100% success rate. The measurements for the maximum diameter of the ostium in the 3D heart model were not significantly different from those obtained via angiography, TEE, and MSCT. Specifically, the maximum diameter of the landing zone did not differ significantly compared to TEE and angiographic measurements (20.90 ± 3.42 mm vs. 20.96 ± 4.81 mm, *p* = 0.563; compared to 21.20 ± 3.90 mm, *p* = 0.291). However, these measurements were significantly smaller than the average MSCT measurements (18.30 ± 2.68 mm vs. 21.03 ± 4.34 mm, *p* < 0.001). Additionally, the predicted implantation angles showed no significant differences between the 3D heart model and MSCT. **Conclusions:** The intraprocedural application of this prototype is both safe and feasible. The measurements obtained from the 3D heart model were consistent with those from TEE and angiography, although discrepancies were noted when compared to MSCT measurements. Notably, the predicted implantation angles demonstrated strong agreement with MSCT, further supporting the prototype’s efficacy in clinical settings.

## 1. Introduction

The minimally invasive treatment of structural heart diseases has evolved rapidly in recent years, marked by an increase in the number, complexity, and variety of procedures aimed at improving patient outcomes [1]. Transcatheter closure of the left atrial appendage (LAAC) has emerged as a reliable stroke prophylaxis for patients with nonvalvular atrial fibrillation who have contraindications to anticoagulant therapy, such as gastrointestinal or cerebral bleeding [2]. Despite this advancement, there remains a significant challenge due to the highly variable anatomy of the left atrial appendage (LAA), which can differ greatly in size and shape among individuals [3].

While four typical LAA morphologies—chicken wing, windsock, cauliflower, and cactus—are commonly described, the occurrence of mixed forms complicates accurate device size selection during procedures [4]. Consequently, there is a pressing need for improved imaging modalities to better evaluate the anatomical features of the LAA. Current options include transesophageal echocardiography (TEE), angiography, multislice computed tomography (MSCT), magnetic resonance imaging, and intracardiac echocardiography [5]. Evidence suggests that MSCT measurements are, on average, 1–3 mm larger than two-dimensional (2D) TEE measurements, 2 mm larger than three-dimensional (3D) TEE measurements, and 2–4 mm larger than fluoroscopic measurements. Moreover, accurate angulation of the C-arm is essential for LAA visualization, device positioning, and successful device release, significantly impacting procedural success [6].

The expert consensus currently recommends TEE or cardiac MSCT for pre-procedural planning [2]. Innovative methods such as 3D printing and virtual reality (VR) are also being explored in this space [7,8]. VR visualization of the LAA using MSCT data has shown feasibility in enabling accurate, reproducible measurements and improving 3D orientation for LAAC planning. Nevertheless, each modality presents specific strengths and weaknesses, and precise sizing requires adequate training and time investment. For intraprocedural guidance, TEE remains critical to visualize cardiac tissue and orient catheters and devices effectively [9]. Recent studies have demonstrated that real-time echocardiography-fluoroscopy fusion imaging (FI) is a safe method that may reduce overall procedure time, time to successful transseptal puncture, and periprocedural contrast volume [10].

In this study, we take a significant step forward by investigating the feasibility, safety, and accuracy of an automated sizing tool that leverages patient-specific 3D heart models for LAA measurements during transcatheter closure. This automatic sizing tool, which is currently in the prototype phase (pFI, EchoNavigator version 3.0, Philips Healthcare), integrates echocardiographic images into fluoroscopic images in real-time, enhancing spatial orientation for interventional cardiologists [11]. The safety and clinical utility of this prototype have previously been validated in the context of transcatheter aortic valve interventions [12]. By addressing the existing research gaps in LAA imaging and device sizing, this study aims to underscore the potential impact of artificial intelligence in streamlining workflow and enhancing procedural accuracy.

## 2. Materials and Methods

### 2.1. Study Design

We conducted clinical, prospective, and monocentric study at the University Hospital Duesseldorf, Germany. We screened 79 patients with nonvalvular atrial fibrillation and contraindication to oral anticoagulation who underwent LAAC and preprocedural MSCT at our heart center between June 2019 and January 2022. Exclusion criteria were a glomerular filtration rate (GFR) < 30 mL/min/1.73 m^2^, atrial or ventricular thrombi, AF related to valvular heart disease, lack of informed consent, and age < 18 years. An Amulet AmplatzerTM closure system was implanted in all patients under conscious sedation and with intraprocedural guidance by TEE and fluoroscopy. The study was performed in line with the Declaration of Helsinki and necessary ethical oversight was secured (IRB approval no. 5272R). All patients provided written informed consent.

### 2.2. Multislice Computed Tomography

Preprocedural high-resolution MSCT was obtained in all patients with a single-source computed tomography scanner (150 ms, 128 × 0.6 mm, SOMATOM Definition Edge, Siemens Healthineers, Erlangen, Germany) triggered by electrogram. GFR < 30 mL/min/1.73 m^2^ was an exclusion for the examination. MSCT acquisition was carried out in accordance with recommendations from the experts. Images were obtained at 30–60% of the R-R interval. A contrast medium of 80 mL was administered to all patients to perform MSCT angiography with bolus tracking. According to established protocols, a delayed scan was performed after contrast injection to achieve optimal contrast distribution. After the successful completion of the examination, the image data sets were saved as Digital Imaging and Communications in Medicine files on the local server, and a dedicated software was used for analysis (3mensio Structural HeartTM 10,0, Pie Medical Imaging BV, Maastricht, The Netherlands). All data sets were evaluated by physicians experienced in this modality (>500 evaluated MSCT data sets). First, the data were evaluated in terms of slow flow, sludge, or LAA thrombi. Next, the measurements were performed, the ostium was defined as the line that derives from the pulmonary vein ridge superiorly to the inferior junction of the LA/LAA at the circumflex artery. The landing zone measurements (widest diameters) were obtained at 10–12 mm within the orifice using the cross-sectional projection. With the help of the software, it was also possible to determine the angulations for the C-arm positioning already preprocedural. These angulations are selected to minimize foreshortening and overlapping of structures, and can be adjusted manually based on operator preference.

### 2.3. Transesophageal Echocardiography

All patients underwent intraprocedural TEE to exclude LAA thrombi, assess LAA morphology in detail, and determine the size of the occluder. TEE measurements were performed with an EPIQ 7 ultrasound machine and ultrasound probe Typ X8 (Philips, Amsterdam, The Netherlands) under conscious sedation by an advanced imaging cardiologist. After volume loading, the TEE probe was positioned in the middle of the esophagus, in slight retroflexion. The measurements were performed at end-systole in at least four views: 0°, 45°, 90°, and 135°. The ostium was defined as a line between the tip of the Coumadin ridge and the circumflex artery, and the landing zone was defined as the line 10–12 mm distal from the ostium perpendicular to the long axis of the LAA. According to the manufacturer’s instructions, the selection of the device size was based on the maximum diameter of the landing zone.

### 2.4. Angiography

Angiographic measurements were intraprocedurally performed after transseptal puncture with contrast administration via a pigtail catheter. After positioning the catheter in the LAA, the projections were recorded in the detector angulations Right Anterior Oblique (RAO) 30/Cranial (CRA) 20 and RAO 30/Caudal (CAU) 20. Again, the ostium and the landing zone were measured.

### 2.5. Fusion Imaging and LAA Sizing Based on a 3D-Heart Model

**Figure 1:**
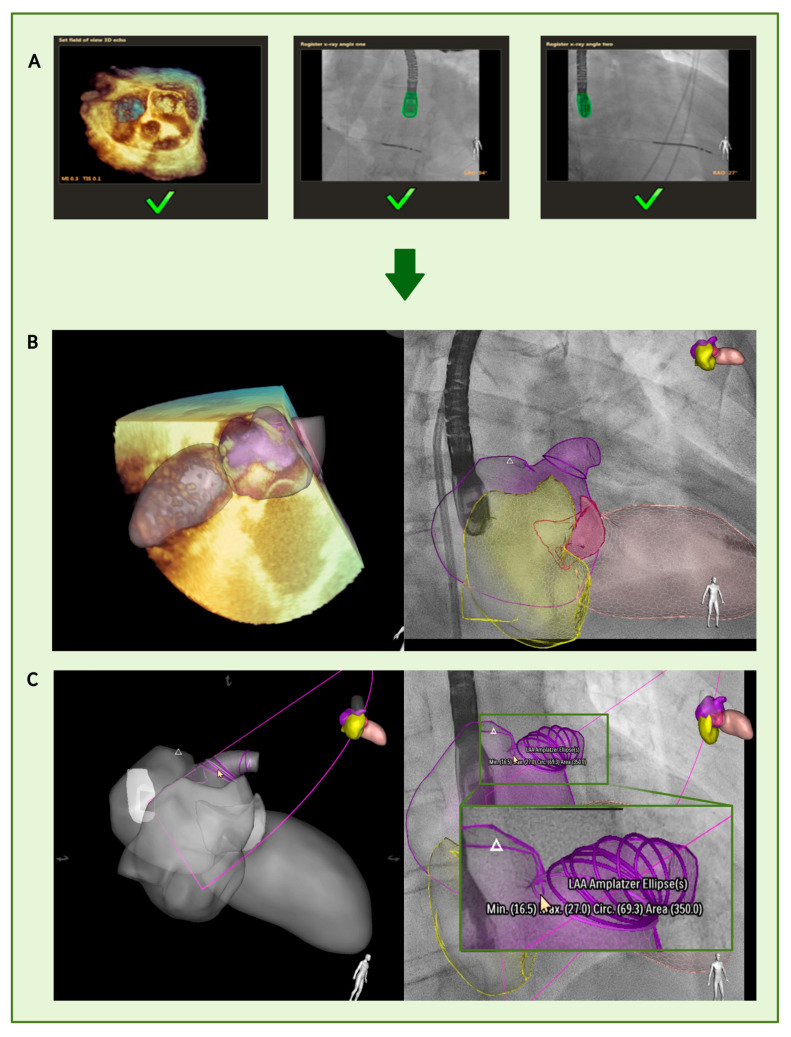
Automatic segmentation and measurements. (**A**): Calibrating the TEE probe for the orientation of the C-arm. (**B**): Establishment of a patient-specific 3D heart model with color marking of the heart structures (LAA purple, right atrium yellow, aortic valve pink, and left chamber light pink). (**C**): In the last step, six ellipses were fitted to the entire LAA to perform the measurements.

The latest development of FI fusion allows the automatic establishment of a patient-specific 3D heart model based on intraprocedural acquired TEE imaging data. In this study, a prototype system was applied during the procedure for the first time. The 3D heart model utilized in this study was developed employing a deep learning algorithm trained on a diverse dataset encompassing various LAA morphologies. The training dataset consisted of a mixture of synthetic 3D models and clinical imaging datasets, ensuring a comprehensive representation of anatomical variations. The model underwent validation using a separate dataset of images not previously used in the training phase, which allowed for an impartial assessment of its performance in accurately recognizing and measuring LAA dimensions. These algorithms can recognize general cardiac morphology, dimensions, and anatomical shape. Once the initial identification is complete, the software automatically detects cardiac boundaries and adjusts them to align with a generic anatomical patient-specific 3D heart model within a few seconds. Following the calibration of the TEE probe relative to the C-arm orientation (Figure 1A), automatic segmentation is performed. This allows for the visualization of various cardiac structures, such as the left atrial appendage (LAA, shown in purple) and the right atrium (shown in yellow), as demonstrated in Figure 1B. The fusion of TEE and fluoroscopic imaging enables the superimposition of soft tissue structures that are not visible on standard fluoroscopy. This fusion significantly enhances procedural guidance by providing accurate anatomical context in real-time, aiding in navigation, device positioning, and avoidance of complications such as inadvertent perforation of the atrial wall. Although fluoroscopy fusion is not strictly required for the automatic segmentation itself, it offers substantial procedural advantages. It allows for the dynamic alignment of the segmented anatomical model with the fluoroscopic environment, enhancing spatial orientation and procedural safety. In addition, the prototype provided a novel capability for fully automated LAA measurements. The surface of the LAA model is mathematically represented by a mesh comprised of interconnected triangular elements. Using this mesh, six ellipses are algorithmically fitted along the entire length of the LAA. Based on these ellipses, the system automatically calculates key anatomical dimensions, such as the minimal and maximal diameters of the ostium and landing zone (as shown in Figure 1C). These measurements are generated without manual input and, in the current version of the prototype, cannot be adjusted or edited by the operator.

### 2.6. Study Endpoints

#### 2.6.1. Feasibility and Accuracy

Feasibility and accuracy of automated sizing in all LAA morphologies and the comparison of standard parameters (ostium and landing zone) with three established imaging modalities, namely 2D TEE, angiography, and MSCT, were assessed. The standard parameters, ostium, and landing zone were collected in millimeters (mm). MSCT examinations were performed preprocedural, whereas automated, echocardiographic, and angiographic measurements were performed intraprocedural. Furthermore, we analyzed the selected device size and the inter-rater reliability of the MSCT measurements and compared the angulations. Angulations were measured preprocedural using the 3mensio software for CT analysis and intraprocedural using the 3D heart model. In all cases, while LAA morphologies presented ambiguities, automated measurements were obtained from the 3D heart model and compared with manual measurements taken by experienced operators using traditional imaging techniques (TEE, MSCT, and angiography). To minimize bias in the image analysis process, blinding was performed during image analysis to ensure that the reviewers analyzing the echocardiographic and CT data were unaware of the clinical outcomes and treatment assignment. This was performed to ensure that the assessments of image quality and anatomical measurements remained objective and free from bias. Furthermore, we assessed which of the remaining rings most closely matched the landing zone dimensions measured using established standard methods. The analysis indicated that ring 3 exhibited the highest agreement.

#### 2.6.2. Efficacy and Safety

Successful implantation and procedural parameters (total procedure time, fluoroscopy time, area–dose product, and contrast agent amount), were analyzed as efficacy endpoints. Successful performance of the procedure was defined as implantation with complete occlusion of the LAA. Total procedure time was defined as the time from venous puncture to completion of TEE with documentation of a good result. Fluoroscopy time was expressed in minutes, whereas the area–dose product, which provides information about the total radiation dose, was expressed in cGy *cm^2^. Periprocedural safety was defined as the occurrence of complications such as pericardial effusion, bleeding, vascular complications (arteriovenous fistula, pseudoaneurysm, arterial stenosis), ischemic and hemorrhagic stroke, dislocation, and arrhythmia. The ISTH/SSC bleeding assessment tool was used to evaluate and assess the severity of bleeding symptoms [13]. All bleeding with a score of four points (blood transfusion, replacement therapy, or desmopressin) was considered.

#### 2.6.3. Antithrombotic Management and Follow-Up

After LAAC, the anticoagulants were discontinued. All patients received aspirin and clopidogrel for six months, followed by aspirin monotherapy. The follow-up period without anticoagulant therapy after the procedure was for a year. A TEE exam was performed after 3 months and a year.

#### 2.6.4. Statistical Analysis

Continuous variables were summarized as mean ± standard deviation, or as median and interquartile range. Categorical variables were summarized as frequency and percentage of a whole. Shapiro–Wilk test was performed to assess normal distribution. Normally distributed data were compared using Student’s *t*-test and for non-normally distributed data the Mann–Whitney U test was used. Pearson’s or Spearman’s correlation was applied to assess the relationships between the methods. A Bland–Altman plot was used to assess agreement between the methods. All tests were two-sided, and *p*-values below 0.05 were considered statistically significant.

To assess the interobserver reliability, two independent experienced imaging specialists performed all MSCT measurements and determined preprocedural angulations. The variables were evaluated by the intraclass correlation coefficient (ICC). Interobserver agreement was interpreted following Koo and Li as excellent for ICC > 0.90, good between 0.75 and 0.90, moderate between 0.50 and 0.75, and poor < 0.50 [14]. All analyses were conducted using IBM SPSS statistics version 28 for Windows [International Business Machines Corporation (IBM^®^ Corp.), Armonk, NY, USA].

## 3. Results

### 3.1. Patient Population

**Figure 2:**
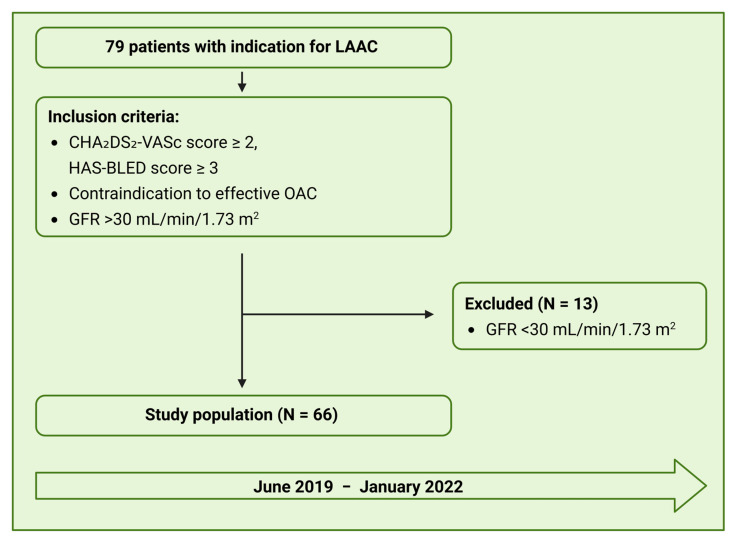
Study flowchart. A total of 79 were screened from June 2019 to January 2022. Due to impaired renal function, 13 patients were excluded, resulting in a study population of 66. GFR—Glomerular filtrations rate; LAAC—Left atrial appendage closure; OAC—Oral anticoagulation.

We prospectively included 66 patients, who successfully underwent LAAC with Amulet device at the Heart Center Duesseldorf, from June 2019 to January 2022 (Figure 2). The mean age of the study population was 75 ± 7 years, and more than half (38 patients, 58%) were male. The most common comorbidities included arterial hypertension (83.3 %), heart failure (75.8%), and coronary artery disease (50.0%). The most common indication for LAAC was an increased risk of bleeding and contraindications to oral anticoagulation, such as chronic kidney disease. Gastrointestinal bleeding was the second most frequent indication. Further baseline characteristics are displayed in Table 1.

### 3.2. Feasibility and Accuracy

#### 3.2.1. Comparison of Ostium Measurements

**Figure 3:**
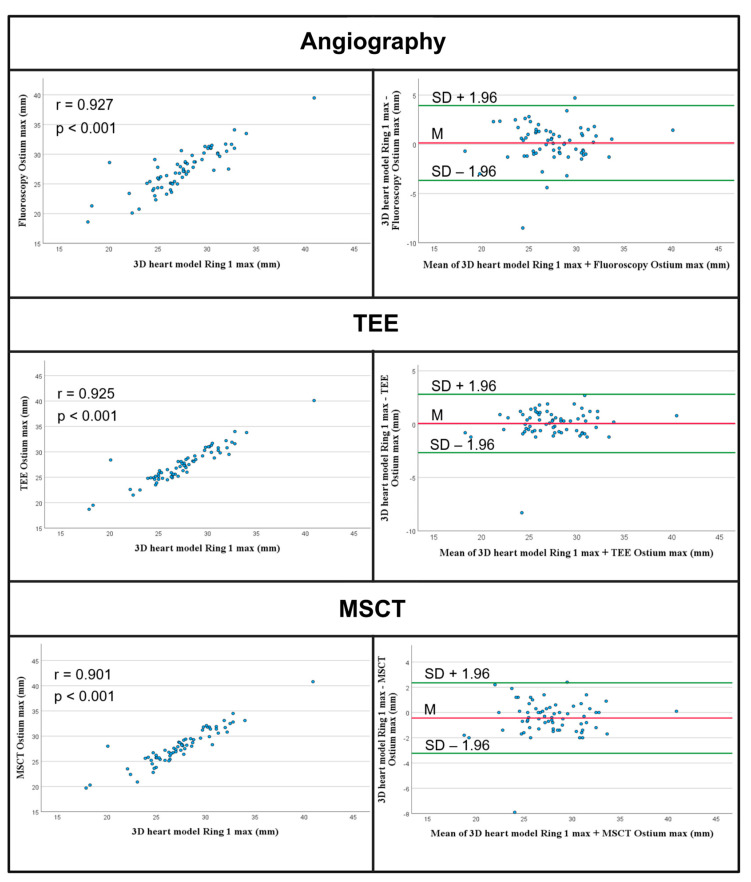
Measurements of the maximum diameter of the ostium. Scatterplots and Bland–Altman plots show strong correlation and agreement of the measurements of the maximum diameter of the ostium. TEE—Transesophageal Echocardiography; MSCT—multislice computed tomography.

The measurements of the maximum diameter of the ostium were 27.54 ± 3.71 and not significantly different from the other modalities; angiography: 27.41 ± 3.59, *p* = 0.239; TEE: 27.47 ± 3.51, *p* = 0.243; and MSCT: 27.98 ± 3.60, *p* = 0.244. Figure 3 presents graphical representations of Pearson correlations and Bland–Altman plots of the measurements of the maximum diameter, and Table 2 presents the results. The measurements of the minimum diameter of the ostium using the 3D heart model were 18.18 ± 2.57 mm and were significantly different from the angiographic (19.97 ± 3.02 mm, *p* < 0.001), echocardiographic (18.94 ± 2.70 mm, *p* < 0.001), and MSCT measurements (20.33 ± 3.35 mm, *p* < 0.001).

#### 3.2.2. Comparison of Landing Zone Measurements

**Figure 4:**
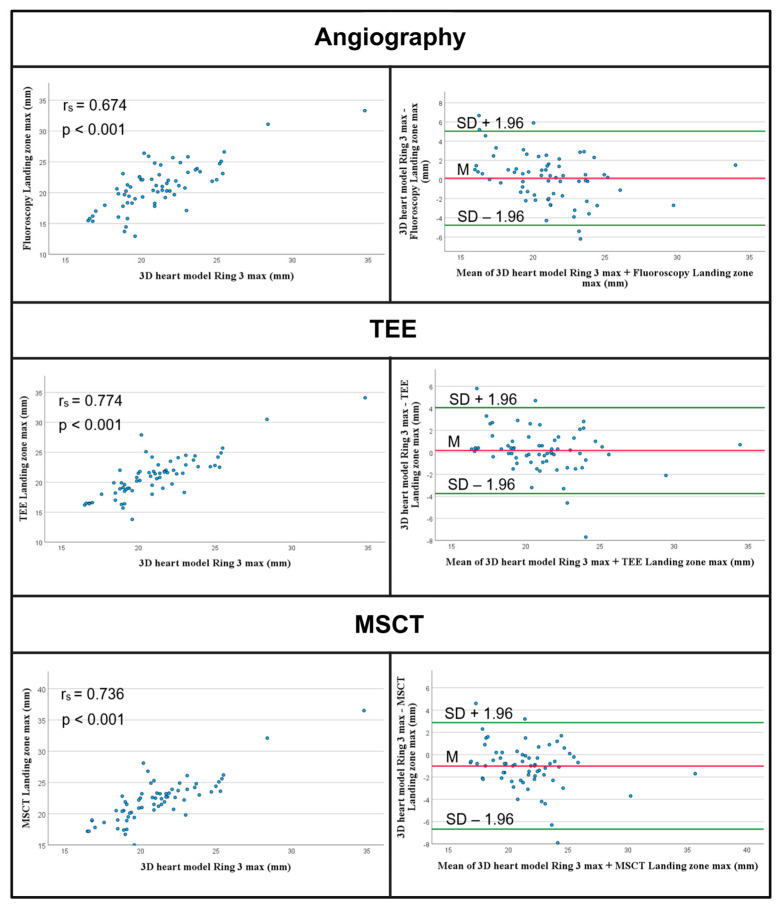
Measurements of the maximum diameter of the landing zone. Scatterplots and Bland–Altman plots show moderate or strong correlation and agreement of the measurements and large dispersion of the data. TEE—Transesophageal Echocardiography; MSCT—multislice computed tomography.

Furthermore, we assessed which of the remaining rings most closely matched the landing zone dimensions measured using established standard methods. The analysis indicated that ring 3 exhibited the highest agreement. The minimum landing zone diameter was determined to be 15.55 ± 2.53 mm, significantly smaller than the diameters measured using angiography (18.18 ± 4.27 mm, *p* < 0.001), echocardiography (17.05 ± 3.22 mm, *p* = 0.004), and MSCT (18.50 ± 4.32 mm, *p* < 0.001). The maximum landing zone diameter for the 3D heart model was recorded at 20.90 ± 3.24 mm, which did not differ significantly from the angiographic (20.96 ± 4.81 mm, *p* = 0.563) and TEE measurements (21.20 ± 3.90 mm, *p* = 0.291). Conversely, the MSCT measurements were significantly larger at 20.96 ± 4.81 mm (*p* < 0.001). Furthermore, the mean MSCT measurements were also significantly greater (18.30 ± 2.68 mm vs. 21.03 ± 4.34 mm, *p* < 0.001) (see Table 3). Spearman correlations and Bland–Altman plots detailing the maximum diameter measurements are presented in Figure 4.

#### 3.2.3. Analysis of Selected Device Size

We further analyzed device selection recommendations based on pFI only and compared them with periprocedural device choice. It was shown that in 86% there was an agreement with the implanted systems.

#### 3.2.4. Analysis of the Angulations

**Figure 5:**
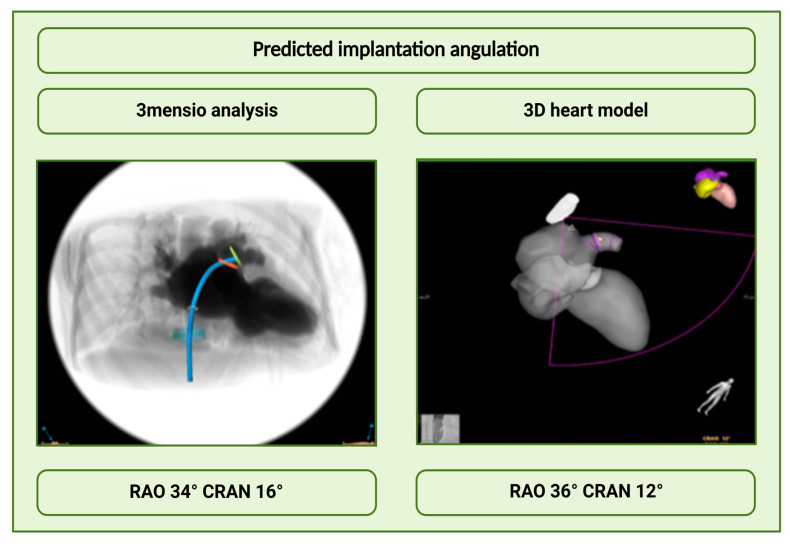
Comparison of angulations. There was a good agreement between the predicted 3mensio angulations and the intraprocedural 3D heart model angulations.

Angulations performed with the 3D heart model and 3mensio software were also compared as part of the analysis (Figure 5). For the analysis, a new variable was created from each of the RAO and CRA/CAU variables that were present in the data set by calculating the average of RAO and CRA or CAU. The *p*-value of the paired samples t-test was greater than 0.05 in both cases, indicating that RAO CRA as well as RAO CAU were not significantly different between the 3D heart model and MSCT. In addition, inter-rater reliability was calculated for angulations measured on MSCT by two experienced cardiologists. For the angulation RAO CRA an inter-rater reliability of 0.953 (*p*-value < 0.001) resulted. Thus, a statistically significant and very high inter-rater reliability was present. For the angulation, RAO CAU showed a value of 0.975 (*p* < 0.001). Again, a statistically significant and high agreement was seen.

#### 3.2.5. Inter-Rater Reliability Analysis of the MSCT Measurements

With a value of 0.857, the agreement of the ostium measurements was very high. For the landing zone, an inter-rater reliability of 0.839 (*p* < 0.001) could be presented. Again, this showed significant and high agreement.

#### 3.2.6. Efficacy and Safety

##### Procedure Parameters

All procedures could be performed successfully. The interventions have lasted on average 87 ± 24.5 min, and the fluoroscopy time was 11.9 ± 10.2 min. The dose–area product was 2088.3 ± 1967.7, and an average of 53.8 ± 27.6 mL of contrast medium was applied.

#### 3.2.7. Procedural Safety

Postprocedural hemorrhage requiring transfusion occurred in three patients. One patient experienced a third-degree intermittent AV block peri interventional. Prolonged post-procedure monitoring revealed sinus rhythm throughout. No other arrhythmogenic events were detected. One patient experienced bradycardia during the procedure. Due to postinterventional persistence, pacemaker implantation was performed. No further complications occurred, and the use of the prototype was not associated with any complications.

## 4. Discussion

The main results of this study can be summarized as follows:

(1) Automated echocardiographic LAA sizing based on a patient-specific 3D heart model prototype in real-time is safe and feasible.
(2) The automated landing zone measurements demonstrated good agreement with the TEE and angiographic measurements, but not with the MSCT measurements.
(3) Intraprocedural implantation angulations performed with the 3D heart model matched very well with the predicted angulations using the 3mensio software.

Precise device sizing for left atrial appendage closure (LAAC) is crucial for ensuring procedural success. Consequently, imaging methods play a vital role and have seen significant advancements in recent years [15]. Various imaging techniques are now employed, and numerous studies have been conducted to define the best method for enhancing preprocedural preparation and intraprocedural safety. While TEE remains the gold standard in many centers, it has inherent limitations, such as invasiveness requiring sedation, dependency on the examiner’s skill, and contraindications in patients with esophageal and gastric conditions. In contrast, multislice computed tomography (MSCT) offers superior image resolution but may not be suitable for all patients, particularly those with impaired kidney function.

The identification of an appropriate device size is often determined by the maximum diameter of the landing zone. However, this approach presents risks of oversizing in appendages exhibiting markedly elliptical shapes. Freixa et al. have proposed that using mean diameters of the LAA may be a more effective sizing strategy, highlighting an important gap in current practices [16]. This underscores the need for the continuous development of imaging methods to facilitate more precise interventions.

As a pioneering heart center, we investigated the periprocedural generated patient-specific 3D heart model for automated LAA segmentations and measurements. We demonstrated that these segmentations could be performed successfully without prolonging the procedure or increasing complications. Our analysis revealed strong correlations in the minimum and maximum diameters of the ostium through statistical evaluation. While maximum diameter measurements were comparable to those obtained via angiography and TEE, we found that measurements of the minimum ostium diameter derived from the 3D heart model were significantly smaller than those from other modalities, indicating potential areas for improvement.

The current prototype utilized a primarily windsock/chicken wing-like morphology, which may not accurately represent diverse patient anatomies and could contribute to suboptimal sizing outcomes. A suboptimal alignment of the device increases the risk of incomplete LAA closure and complications such as a peri-device leak [17]. An option to manually adjust ring placement could enhance sizing accuracy and reduce the risk of inadequate LAA closure. Previous findings during transcatheter aortic valve implantation have shown that prototype-based annular measurements were significantly larger than MSCT assessments, with only 61% agreement with implanted devices [12].

Although the use of the 3D heart model resulted in shorter procedure times, it did not yield significant differences in contrast agent usage or fluoroscopy durations. In conclusion, our findings underscore that LAA ostium diameter assessed through 3D-based cardiac models demonstrates high predictability and agreement with traditional 2D modalities, such as TEE and angiography. We found that while TEE measurements for the maximum diameter of the ostium were consistent with those obtained through angiography, statistically significant differences were observed in the minimum diameter measurements across various modalities. This suggests that TEE may not perform equally well for all types of LAA configurations, particularly about measuring the minimum diameter, especially in reversed chicken wing morphologies. However, when examining landing zone diameter—critical for device sizing—the convergence between 3D models and MSCT measurements reveals persistent discrepancies. This highlights the dimensional accuracy limitations between 2D and 3D imaging modalities and emphasizes the need for multi-source averaging to obtain a comprehensive assessment for procedural planning.

Furthermore, we evaluated the predicted implantation angulation by the prototype in comparison to MSCT and found a strong correlation, indicating feasibility in predicting implantation angles. However, the current state of automatic measurement methods for the LAA remains underexplored. A retrospective analysis by Morais et al. demonstrated good agreement between automatic and manual measurements of LAA dimensions based on 3D echocardiographic images [18]. Notably, the discrepancy in our study may stem from the simplified representation of the LAA, as only one morphology (windsock/chicken wing-like shape) was analyzed. A broader and more precise structural representation of the LAA may improve measurement accuracy.

Similar studies have also employed AI-driven methodologies for LAA measurements. For instance, Sun et al. showcased an AI software solution that effectively achieved high reproducibility in standard parameter collection, demonstrating compatibility with CT assessments [19]. In our study, while automated measurements were based on echocardiographic data, MSCT could also facilitate similar evaluations. Additional research by Michielis et al. using AI-based MSCT analysis to plan LAAC revealed comparable results between automated and manual measurements, along with optimized workflow duration [20]. Moreover, the FEops HEARTguide offers AI-assisted procedural simulations based on MSCT data, showing potential for enhanced procedural efficiency, as highlighted in the PREDICT-LAA trial [21,22,23].

This multicenter study indicates that the integration of innovative methods could improve procedural efficiency and outcomes, reinforcing that patient-specific imaging represents a promising approach to optimize LAA sizing. However, further development is essential to enhance measurement precision and maximize procedural planning utility, ultimately benefiting patient safety.

### Limitations

The evaluated 3D heart model is a prototype. As such, this study primarily aimed to validate the applicability and measurement accuracy of this technology. Current limitations include the inability of the model to accurately reflect the full spectrum of LAA morphologies. Additionally, the relatively small sample size restricts generalizability to the broader population. Future advancements in the prototype, alongside studies involving larger cohorts, are necessary to refine measurements and expand its application to other procedures in structural heart disease.

The evaluated 3D heart model represents a prototype; therefore, this study primarily focused on validating the applicability and accuracy of the measurements. The model is currently not capable of accurately representing the diverse morphologies of the left atrial appendage (LAA), which limits its effectiveness in various clinical scenarios. Additionally, the relatively small sample size restricts the generalizability of the findings to the broader patient population. Any potential confounders, patient selection biases, or missing data could impact the results and conclusions drawn from this study. Furthermore, there exists a risk of overfitting or dataset dependence related to the application of artificial intelligence (AI) in automated measurements.

Future developments of the prototype, as well as studies involving larger and more diverse subject cohorts, are essential to enhance measurement accuracy and expand its application to additional procedures within the realm of structural heart disease. Continuous refinement will be necessary to address these limitations and ensure the safe and effective integration of this innovative imaging technology into clinical practice

## 5. Conclusions

The study demonstrated that the periprocedural application of a 3D heart model derived from echocardiographic image datasets is both safe and efficient. Intraprocedural automatic measurements of the left atrial appendage (LAA) displayed good correlation with transesophageal echocardiography (TEE) and angiographic measurements; however, the values at the landing zone were significantly smaller when compared to multidetector computed tomography (MSCT). Additionally, the prototype effectively predicts implantation angles. To further enhance the accuracy and utility of this innovative approach, it is essential to prioritize the development of the model to improve its performance and ensure its safe application in future structural interventions.

We recommend that future studies adopt a multicenter approach to validate the findings across diverse patient populations and clinical settings. This will provide a more comprehensive understanding of the model’s effectiveness and its implementation in routine practice. Furthermore, it should be considered to incorporate advanced imaging technologies and AI capabilities in procedural planning to achieve better outcomes, as precise imaging and measurement of cardiac and extracardiac structures are paramount for successful interventional procedures.

Factors such as current data availability, advancements in imaging technology, and the incorporation of artificial intelligence in interventional cardiology underscore the importance of precise imaging and measurement of cardiac and extracardiac structures for interventionalists to accurately plan and execute procedures safely.

## Figures and Tables

**Table 1:** Baseline characteristics.

Patient Characteristics	N = 66
Male gender, *n* (%)	38 (57.6%)
Age, M (years) ± SD	75 ± 7
Height, M (m) ± SD	1.73 ± 0.1
Weight, M (kg) ± SD	77 ± 23
BMI, M (kg/m^2^) ± SD	26 ± 6
CHA2DS2-VASc-Score, M ± SD	4 ± 2
HAS-BLED-Score, M ± SD	3 ± 2
Atrial fibrillation, *n* (%)	66 (100)
Paroxysmal, *n* (%)	39 (59.1)
Persistent, *n* (%)	6 (9.1)
Permanent, *n* (%)	21 (31.8)
Arterial hypertension, *n* (%)	55 (83.3)
Heart failure, *n* (%)	50 (75.8)
Coronary artery disease, *n* (%)	33 (50.0)
s/p. MI, *n* (%)	6 (9.1)
s/p. CABG, *n* (%)	9 (13.6)
s/p PCI, *n* (%)	24 (36.4)
Chronic kidney disease, *n* (%)	22 (33.3)
Diabetes mellitus, *n* (%)	30 (45.5)
Morphology	
Chicken Wing, *n* (%)	42 (62.6)
Cactus, *n* (%)	10 (15.2)
Windsock, *n* (%)	12 (18.2)
Cauliflower, *n* (%)	2 (3)
Indication for LAAC	
Contraindications to OAC	
Increased risk of bleeding, *n* (%)	26 (39.4 %)
Gastrointestinal bleeding, *n* (%)	19 (28.8 %)
Other bleeding, *n* (%)	17 (25.8 %)
INR instability, *n*(%)	4 (6.1 %)

**Table 2:** Measurements of the minimum and maximum diameter of the ostium.

Variable	M ± SD	*p*-Value
3D heart model Ring 1 min, M (mm) ± SD	18.18 ± 2.57	
Angiography, M (mm) ± SD	19.97 ± 3.02	*p* < 0.001
TEE, M (mm) ± SD	18.94 ± 2.70	*p* < 0.001
MSCT, M (mm) ± SD	20.33 ± 3.35	*p* < 0.001
3D heart model Ring 1 max, M (mm) ± SD	27.54 ± 3.71	
Angiography, M (mm) ± SD	27.41 ± 3.59	0.239
TEE, M (mm) ± SD	27.47 ± 3.51	0.243
MSCT, M (mm) ± SD	27.98 ± 3.60	0.244

There were no significant differences between measurements of the maximum diameter of the ostium. TEE—Transesophageal Echocardiography; MSCT—multislice computed tomography.

**Table 3:** Measurements of the maximum diameter of the landing zone.

Variable	M ± IQR	*p*-Wert
Ring 2		
3D heart model Ring 2 max, M (mm) ± IQR	23.10 ± 3.65	
Angiography, M (mm) ± IQR	20.96 ± 4.81	*p* < 0.001
TEE, M (mm) ± IQR	21.20 ± 3.90	*p* < 0.001
MSCT, M (mm) ± IQR	22.15 ± 3.49	*p* < 0.001
Ring 3		
3D heart model Ring 3 max, M (mm) ± IQR	20.90 ± 3.42	
Angiography, M (mm) ± IQR	20.96 ± 4.81	0.563
TEE, M (mm) ± IQR	21.20 ± 3.90	0.291
MSCT, M (mm) ± IQR	22.15 ± 3.49	*p* < 0.001
Ring 4		
3D heart model Ring 4 max, M (mm) ± IQR	20.10 ± 3.35	
Angiography, M (mm) ± IQR	20.96 ± 4.81	0.010
TEE, M (mm) ± IQR	21.20 ± 3.90	*p* < 0.001
MSCT, M (mm) ± IQR	22.15 ± 3.49	*p* < 0.001
Ring 5		
3D heart model Ring 5 max, M (mm) ± IQR	18.90 ± 3.60	
Angiography, M (mm) ± IQR	20.96 ± 4.81	*p* < 0.001
TEE, M (mm) ± IQR	21.20 ± 3.90	*p* < 0.001
MSCT, M (mm) ± IQR	22.15 ± 3.49	*p* < 0.001
Ring 6		
3D heart model Ring 6 max, M (mm) ± IQR	17.60 ± 3.20	
Angiography, M (mm) ± IQR	20.96 ± 4.81	*p* < 0.001
TEE, M (mm) ± IQR	21.20 ± 3.90	*p* < 0.001
MSCT, M (mm) ± IQR	22.15 ± 3.49	*p* < 0.001

The analysis of the remaining rings showed the best agreement between ring 3 and the measurements of the maximal diameter of the landing zone collected by established methods. TEE—Transesophageal Echocardiography; MSCT—multislice computed tomography.

## Data Availability

The anonymized data can be requested from the authors if required.

## Abbreviations

The following abbreviations are used in this manuscript:

2D	Two dimensional
3D	Three dimensional
AI	Artificial intelligence
CRA	Cranial
CAU	Caudal
FI	Fusion imaging
GFR	Glomerular filtration rate
ICC	Intraclass correlation coefficient
LAA	Left atrial appendage
LAAC	Left atrial appendage closure
MRI	Magnetic resonance imaging
MSCT	Multislice computed tomography
RAO	Right anterior oblique
TEE	Transesophageal echocardiography
